# The role of circulating tumor DNA in gynecological cancer management

**DOI:** 10.3389/fonc.2025.1664172

**Published:** 2026-01-12

**Authors:** Min Gao, Pei-Yan Yu, Run-Xuan Li, Chen Chen, Xiao-Feng Cong, Zi-Ling Liu

**Affiliations:** Cancer Center, the First Hospital of Jilin University, Changchun, Jilin, China

**Keywords:** circulating tumor DNA, gynecological cancer, next-generation sequencing, polymerase chain reaction, precision oncology

## Abstract

Ovarian cancer, endometrial cancer, and cervical cancer represent the three most prevalent primary gynecological tumors that pose a significant threat to women’s health globally. To enhance survival rates for patients with gynecological cancers, there is an urgent need to optimize disease detection and monitoring technologies aimed at improving early diagnosis, treatment efficacy monitoring, treatment guidance, and prognosis prediction. Currently, traditional methods for identifying and tracking malignant tumors primarily depend on imaging studies, supplemented by blood-based protein biomarker testing. However, these biomarkers generally exhibit limitations in terms of sensitivity and specificity. Circulating tumor DNA (ctDNA), comprising DNA fragments released by cancer cells into the bloodstream, can be detected using liquid biopsy technology. Compared to tissue biopsy, ctDNA testing offers the advantages of minimal invasiveness and continuous monitoring, thereby eliminating the need for multiple surgeries. In addition, it may detect disease recurrence or predict behavior in ways that entity organization biopsy, tumor marker monitoring, and imaging cannot. Increasing evidence indicates that ctDNA has the potential to enhance the clinical management of gynecological tumors by improving early diagnosis, monitoring treatment responses, detecting recurrences, and predicting prognosis. This review aims to summarize the application of ctDNA in gynecological cancers and provide an overview of its comprehensive research and clinical validation. Furthermore, we discuss future development directions based on existing challenges and identify areas requiring further research to elucidate the potential of ctDNA in clinical applications.

## 1 Introduction

Globally, gynecological malignancies pose a serious threat to women’s health and well-being. According to data released by the International Agency for Research on Cancer (IARC) in 2022, approximately 1.33 million new cases of gynecological malignancies are diagnosed worldwide each year, resulting in approximately 540,000 deaths (1), with a significant disease burden. Among gynecological malignancies, ovarian cancer, cervical cancer, and endometrial cancer are the three most common types. In the management of gynecological malignancies, early diagnosis, assessment of treatment efficacy, early identification of tumor recurrence and metastasis, and prognosis evaluation are of critical importance (2–4). In these stages, the identification of effective and reliable tumor markers is of paramount importance. Currently, the diagnostic and therapeutic strategies for gynecological tumors face numerous challenges, including the insufficient sensitivity of traditional biomarkers, the invasiveness of tissue biopsies, and sampling biases. Therefore, there is an urgent need to develop novel non-invasive monitoring methods to address these challenges in the diagnosis and treatment of gynecological tumors.

**Figure 1:**
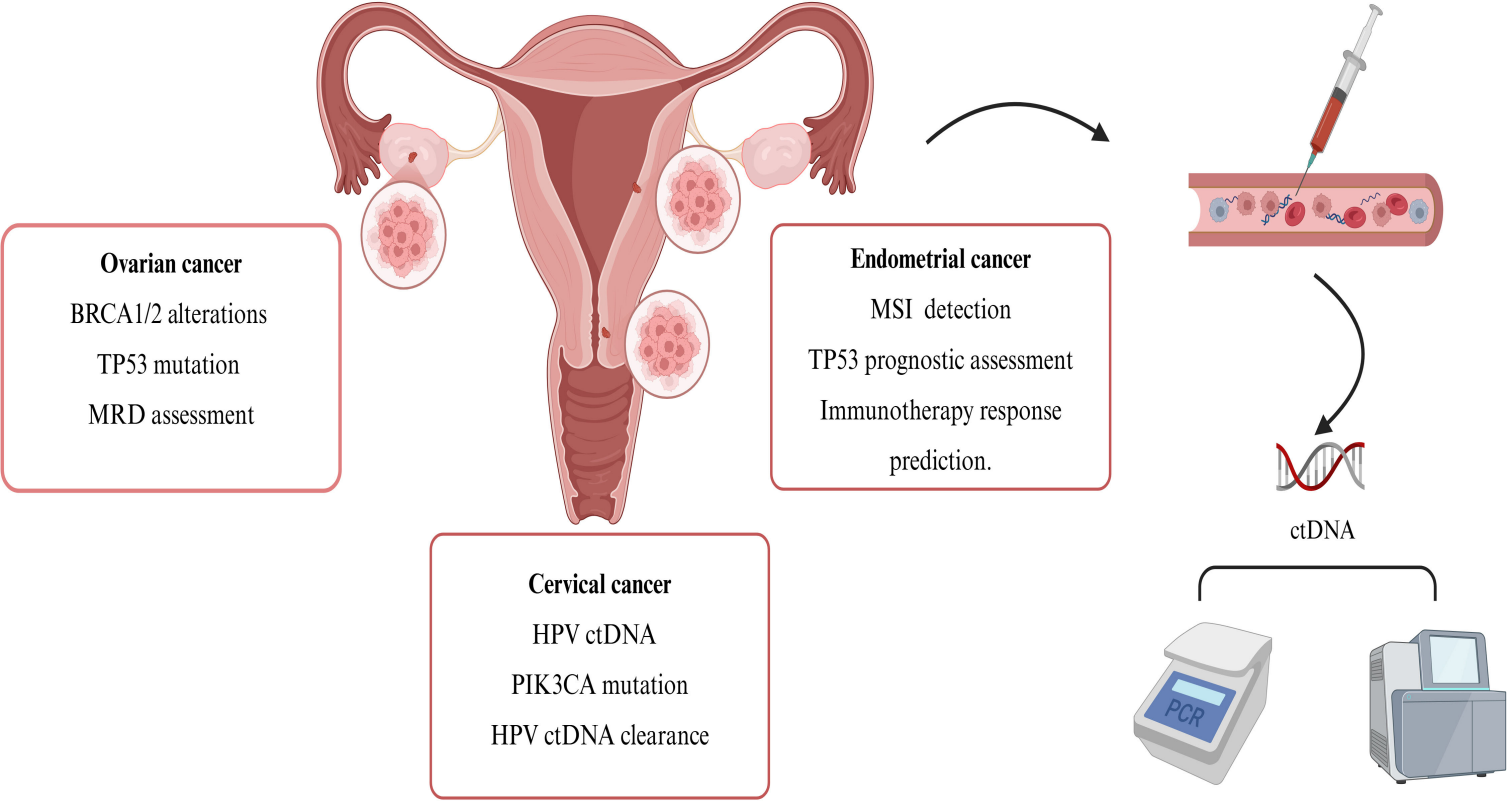
Application of ctDNA analysis in gynecological tumors. Gynecological malignancies, including both primary and metastatic lesions, release ctDNA into the peripheral blood circulation through mechanisms such as cell necrosis, apoptosis, or active secretion. The detection of ctDNA primarily relies on PCR and NGS technologies, which enable precise diagnosis and treatment through the analysis of tumor-specific molecular markers. The application of ctDNA detection in gynecological tumors is becoming increasingly widespread. In ovarian cancer, focus is placed on testing for *BRCA*1/2 germline and somatic mutations to guide PARP inhibitor therapy. Additionally, dynamic monitoring of *TP53* mutations is employed to assess tumor evolution, and ctDNA-based MRD monitoring is utilized to predict recurrence risk. For cervical cancer, HPV-ctDNA quantitative analysis is employed for monitoring virus-associated tumor burden. Furthermore, integrating *PIK3CA* mutation profiles aids in guiding targeted therapy strategies, while dynamic evaluation of prognosis is conducted through ctDNA mutation clearance rates. In endometrial cancer, screening for populations that may benefit from immunotherapy is based on MSI status, and *TP53* mutation testing is incorporated to assess prognosis. ctDNA, circulating tumor DNA; MRD, minimal residual disease; Created in https://BioRender.com.

In this context, liquid biopsy (LB) has emerged as a promising non-invasive diagnostic tool for detecting tumor-derived biomarkers. Liquid biopsy utilizes DNA detection technology to extract tumor-related information from blood samples. Compared to tissue biopsy, blood-based biomarkers present a lower risk of invasiveness to patients and can offer more comprehensive insights into tumor heterogeneity. ctDNA, one of the primary targets of liquid biopsy technology, refers to free DNA fragments released into the peripheral blood by tumor cells through processes such as necrosis or apoptosis. These fragments carry tumor-specific gene mutations and epigenetic features, effectively reflecting the genomic heterogeneity of solid tumors (5). By integrating high-sensitivity methods such as next-generation sequencing (NGS), real-time fluorescent quantitative polymerase chain reaction (RT-qPCR), digital PCR (dPCR), and amplification refractory mutation system (ARMS), ctDNA can be analyzed both qualitatively and quantitatively. These technologies facilitate dynamic assessments of tumor burden, real-time monitoring of treatment responses, and detection of minimal residual disease, thereby offering greater efficiency and accuracy compared to other serum-based markers (6). Currently, ctDNA has garnered substantial research evidence in clinical applications, including early diagnosis, efficacy monitoring, treatment guidance, and prognosis assessment in gynecological tumors, demonstrating significant clinical value. Therefore, this article presents a concise review of the latest advancements in ctDNA detection technology and its clinical applications in gynecological tumors (**Figure 1**, **Table 1**).

**Table 1:** Clinical Applications of ctDNA in Gynecological Tumors. .

Cancer type	Application	Detection methods	Approach	Highest evidence level	Key studies/description	Core finding	Clinical significance	Ref
Ovarian Cancer	Early diagnosis	MethylLight PCR	ctDNA mutations/methylation	Level I	Cohen et al. (2016): NGS study on 32 patients and 32 controls, showing genomic copy number variations in ctDNA.	It can effectively identify early-stage lesions, and combining with traditional markers can enhance diagnostic accuracy	Enhancing early detection rates with auxiliary traditional markers.	(7–16)
	​Efficacy monitoring	NGS/dPCR	*TP53* mutation monitoring	Level II	Hou et al. (2022): Prospective study on ctDNA dynamics during treatment, demonstrating superiority over CA125.	The variation in mutation frequency is significantly correlated with treatment response, and the extent of postoperative clearance reflects the efficacy of tumor reduction	Dynamically assess the efficacy of chemotherapy/surgery.	(17–25)
	Treatment guidelines	NGS	*BRCA1/2* reversion mutations	Level III	Lin et al. (2019): Retrospective analysis of 78 patients, identifying *BRCA* reversal mutations guiding PARP inhibitor therapy.	Patients with reverse mutations have a poor prognosis, indicating resistance to platinum/PARPi but sensitivity to specific targeted therapies	Optimization Basis for Targeted Therapy Post Drug Resistance.	(26–34)
	​​Prognosis evaluation​​	NGS	ctDNA mutations /MRD status	Level II	Wang J et al. (2024): Serial ctDNA testing in 23 patients, 91% sensitivity in predicting recurrence.	Postoperative ctDNA negativity indicates an extremely low risk of recurrence; patients with positive ctDNA have a significantly increased risk of recurrence	MRD status serves as the optimal predictor for recurrence, guiding treatment decisions.	(35–38)
​​Cervical Cancer​​	​​Early diagnosis​​	ddPCR/qPCR	HPV ctDNA	Level II	Gu et al. (2020): Meta-analysis showing HPV ctDNA with high specificity (94%) for cervical cancer diagnosis.	Exhibits high specificity and is associated with the degree of tumor progression	Alternative genomic analysis methods can be considered when tissue samples are insufficient. (2022 NCCN Guidelines).	(39–43)
	​Efficacy monitoring	NGS	ctDNA mutations	Level III	Kim et al. (2023): Study on 25 patients using NGS for HPV ctDNA, comparing with traditional markers.	Mutation status is associated with treatment resistance and survival outcomes	Real-time monitoring of neoadjuvant chemotherapy response and drug resistance.	(44–46)
	​​ Treatment guidelines​​	ddPCR	ctDNA mutations	Level II	KEYNOTE-158 trial (Marabelle et al., 2020): Phase II study on PD-1 inhibitors in MSI-H tumors, guiding immunotherapy.	The plasma mutation profile shows high concordance with tissue; ctDNA-guided therapy can improve survival	Guiding the selection of targeted drugs and adjustment of radiotherapy and chemotherapy regimens.	(47–49)
	​​Prognosis evaluation​​	ddPCR	HPV ctDNA clearance	Level I	CALLA study (Mayadev et al., 2025): Phase III trial showing ctDNA as a prognostic tool for MRD monitoring in LACC.	Patients who achieved clearance after treatment showed significant improvement in prognosis, while those with residual disease had an increased risk of recurrence	Independent prognostic marker, applicable for risk stratification.	(50–54)
​​Endometrial Cancer​​	​​Early diagnosis​​	NGS	ctDNA mutations	Level III	Feng et al. (2021): Retrospective study on ctDNA mutations in high-risk endometrial cancer.	Capable of identifying early-stage lesions, and enhances screening efficacy when combined with other biomarkers	High-risk population screening tool.	(55–60)
	​Efficacy monitoring	NGS	ctDNA load dynamics	Level II	Ashley et al. (2023): Prospective study on ctDNA levels correlating with treatment response and recurrence.	Level changes are associated with tumor burden and treatment response	Monitor treatment response and disease progression.	(61–64)
	​​ Treatment guidelines​​	NGS	ctDNA mutations	Level I	DUO-E trial (Westin et al., 2024): Phase III study on durvalumab and olaparib, showing ctDNA clearance guiding therapy.	Guiding the applicability of immune checkpoint inhibitors; enhancing clearance rates through combined targeted therapy	Biomarkers for immunotherapy.	(65–69)
	​​Prognosis evaluation​​	NGS	*TP53* mutations / ctDNA mutations	Level III	Pereira et al. (2015): Retrospective analysis of ctDNA for predicting recurrence in endometrial cancer.	A positive status significantly indicates a risk of recurrence; patients with *TP53* mutations and co-mutations have a worse prognosis	Superior to traditional markers, guiding stratified therapy.	(3, 55, 66, 70–73)

## 2 ctDNA

### 2.1 Definition and characteristics of ctDNA

ctDNA carries genetic mutations and can be identified in matched tumors from individuals which are specific to the tumor. ctDNA is a component of circulating free DNA (cfDNA) that enters the bloodstream of cancer patients, exhibiting a half-life of less than two hours (74). Comprising short DNA fragments typically ranging from 150 to 200 base pairs (75). Epigenetic or genetic changes, such as tumor-specific methylation markers, rearrangements, copy number variations, and somatic point mutations, allow for the distinction of ctDNA from normal cfDNA fragments (76). The modifications observed in the genetic and epigenetic profiles of ctDNA can mirror the genomic or epigenomic traits of the cells from which they are derived. This characteristic enhances their application in assessing prognosis, identifying residual disease, selecting treatment strategies, and predicting the risks of potential recurrence (77).Studies indicate that the ctDNA content within total cfDNA detected in patient plasma can be as low as 0.01% (78). Consequently, methods employed to detect and characterize ctDNA in cfDNA must possess extremely high sensitivity. Furthermore, the quantity of ctDNA in patient serum varies not only between individuals but also according to tumor type.

### 2.2 Methods for detecting ctDNA

#### 2.2.1 PCR and NGS

Currently, the primary methods for ctDNA detection include PCR analysis and NGS-based technologies. dPCR and droplet digital PCR (ddPCR), which are considered third-generation PCR technologies, have demonstrated high specificity (81%) and exceptional sensitivity (99%) in detecting specific gene mutations in gynecological tumors (79). These technologies can accurately quantify nucleic acids and utilize fluorescent probes to analyze specific mutations or wild-type sequences in biological materials. In contrast, whole-exome sequencing and whole-genome sequencing can simultaneously analyze gene mutations across multiple genomic regions with high sensitivity in a single test. These methods have been widely applied in DNA mutation analysis and the assessment of tumor mutation burden. A recent study conducted a retrospective analysis of gynecological cancer patients using ctDNA analysis, revealing that the majority of patients (75%) exhibited at least one characteristic alteration in their ctDNA. The most common alterations identified were TP53 (56% of patients), PIK3CA (25% of patients), KRAS (13% of patients), and BRAF (10% of patients) (47). Unlike dPCR, target-based NGS can not only identify known mutations but also discover unknown mutations at a relatively low cost; however, its sensitivity to chromosomal copy number alterations and rearrangements is comparatively lower (80). Furthermore, other detection methods such as whole-genome sequencing (WGS) and cancer personalized analysis via deep sequencing (CAPP-Seq) also employ NGS technology to analyze ctDNA in gynecological tumors (81).

#### 2.2.2 Methylation Profiling

The methylation analysis of ctDNA has been extensively utilized in cancer diagnosis and monitoring. Numerous studies indicate that the DNA methylation patterns in ctDNA possess significant value for early detection, treatment evaluation, and personalized medicine. Bi Wang et al. proposed that the methylation status of ctDNA in the blood of OC patients may facilitate early detection and tailored treatment approaches (82). Furthermore, a recent study conducted by Rafael and colleagues has shown that HPV-DNA methylation analysis demonstrates high sensitivity and specificity in the detection of cervical cancer, thereby further substantiating the potential of DNA methylation analysis for early cancer detection (83).

#### 2.2.3 Fragmentomics analysis

The fragmentomics analysis of cfDNA and ctDNA is essential for elucidating the fragmentation characteristics of cfDNA, including its size distribution and biochemical properties at the free ends. Compared to cfDNA, ctDNA fragments are typically shorter (84). Research demonstrates that fragmentomics analysis can yield comprehensive insights into the entire ctDNA population, revealing both the tissue of origin and the pathological features associated with alterations in the fragmentome profile (85–87). Furthermore, cfDNA fragmentomics significantly contributes to differentiating cancer-derived cfDNA, identifying the tissue of origin, and facilitating the early detection of cancer recurrence (88, 89).

#### 2.2.4 Multimodal methods

Multimodal analytical approaches are employed, encompassing the examination of copy number alterations (CNA), as well as epigenetic, genomic, and fragmentomic analyses of cfDNA samples. Recent research indicates that the mechanisms underlying microRNA dysregulation primarily involve the amplification or deletion of microRNA genes, epigenetic modifications, and aberrant transcription factor activity. Notably, miRNA-21, a microRNA extensively studied in the literature, has been demonstrated to be upregulated in various malignant tumors, including breast cancer, lung cancer, and colorectal cancer (90). Parikh et al. found that the integration of epigenomic features could enhance the sensitivity of recurrence detection by 25-36% compared to the use of genomic alterations alone (91).

These methods have a wide range of applications, including the assessment of tumor mutation burden, the identification of epigenetic changes, and the diagnosis or detection of drug-resistant mutations. In the field of gynecological tumors, various methods have been employed to detect and analyze ctDNA, demonstrating high levels of diagnostic sensitivity and specificity. With ongoing technological advancements and the deepening of related research, the standardization and normalization of ctDNA detection will emerge as a key focus for future development.

## 3 Current state of ctDNA research

Currently, ctDNA primarily emphasizes early diagnosis, treatment response monitoring, and prognosis assessment for lung cancer, colorectal cancer, and breast cancer. It plays a crucial role in monitoring minimal residual disease (MRD) following curative treatment (92). MRD refers to the persistent presence of a small number of tumor cells during or after treatment, which are typically undetectable by conventional radiological techniques but can be identified through ctDNA analysis. The clinical value of ctDNA MRD monitoring is multifaceted (93). First, it allows for the early detection of residual lesions, providing an opportunity for timely intervention and potentially improving patient prognosis. Second, ctDNA MRD monitoring serves as a vital tool for evaluating the efficacy of neoadjuvant therapy (NAT). By detecting changes in ctDNA levels in the blood before and during treatment, the effectiveness of the therapy in reducing tumor burden can be assessed. Finally, it holds significant predictive value in evaluating the risk of disease recurrence post-surgery. By monitoring ctDNA levels after surgery, risk stratification can be performed, thereby enhancing the formulation of adjuvant chemotherapy and subsequent treatment plans.

Current clinical research across lung, breast, and colorectal cancers consistently demonstrates the critical role of ctDNA analysis in predicting treatment response and monitoring disease recurrence. In lung cancer, a strong correlation exists between ctDNA clearance and pathological complete response (pCR) or major pathological response (MPR) following neoadjuvant therapy. The presence of MRD post-surgery, as detected via ctDNA, is a significant predictor of poorer disease-free survival (DFS). Furthermore, adjuvant therapies such as atezolizumab or osimertinib significantly improve outcomes and enhance ctDNA clearance, with ctDNA detection often preceding radiographic recurrence by several months (94–99). In breast cancer, ctDNA dynamics during and after neoadjuvant therapy are htivityighly prognostic; a reduction or clearance of ctDNA predicts higher pCR rates and longer DFS, whereas persistent ctDNA post-therapy indicates an increased risk of recurrence. Serial monitoring during adjuvant therapy identifies high-risk patients with high sensitivity (93-100%), and ctDNA positivity frequently precedes clinical recurrence by 7 to 16 months, thereby providing a crucial window for intervention (100–109). Similarly, in colorectal cancer, ctDNA levels accurately reflect tumor burden and response to neoadjuvant chemoradiotherapy (nCRT). Postoperative ctDNA MRD is strongly associated with recurrence risk and poor prognosis, and ctDNA-based surveillance detects recurrence significantly earlier (by an average of 8.7 months) than standard imaging (110–116).

In the field of gynecological cancers, research has predominantly concentrated on the application of ctDNA analysis for disease monitoring, MRD detection, and the identification of specific genetic alterations. However, the optimal timing for its application, the determination of treatment regimens (such as therapy downgrading), and its impact on patient prognosis still necessitate further validation through prospective clinical studies.

## 4 Application of ctDNA in ovarian cancer

### 4.1 Early diagnosis

Ovarian cancer is frequently diagnosed at an advanced stage due to its subtle symptoms, with approximately 70% of patients receiving a diagnosis only in the later stages, which contributes to a poor prognosis (117). Thus, early and accurate diagnosis is essential for effective treatment of ovarian cancer. Currently, the FDA has approved only two tumor markers, CA-125 and human epididymis protein 4 (HE-4), for diagnostic purposes; however, their sensitivity and specificity are relatively low, rendering them inadequate for early screening. With the ongoing advancements in molecular biology technologies, research on the application of ctDNA in the early diagnosis of ovarian cancer has been on the rise.

In ovarian malignant tumors, ctDNA serves as a crucial biomarker for tumor growth, metastasis, treatment response, and recurrence (118). Research indicates that tracking the temporal heterogeneity of ctDNA allows for the determination of evolutionary pathways in ovarian cancer, even at resolutions below the exome scale (119). The current research focuses on multiple ctDNA analysis strategies. During tumorigenesis and progression, hypermethylation of CpG islands within gene promoters can suppress transcription, silence tumor suppressor genes, and activate oncogenes, ultimately promoting malignant tumor transformation (120). DNA methylation represents an early event in tumorigenesis, and the analysis of ctDNA methylation patterns has emerged as a promising method for diagnosing early-stage ovarian cancer (7). However, the clinical utility of individual studies must be evaluated in the context of their validation status and technical reproducibility.

Widschwendter et al. (8) investigated the application of ctDNA in the early diagnosis of ovarian cancer. By monitoring the methylation status of *COL23A1*, *C2CD4D*, and WNT6, the researchers found that the diagnostic rate for early-stage ovarian cancer was 23%, compared to 15.4% for the traditional CA125 testing method, with a specificity of 97%. Singh et al. (9) employed the MethyLight method to analyze 120 tissue samples (85 tumor samples and 35 healthy controls) and 70 matched serum samples (45 tumor samples and 25 healthy controls). The study found that promoter methylation of the tumor suppressor genes *HOXA9* and *HIC1* in ctDNA effectively distinguished epithelial ovarian cancer from healthy controls, yielding an area under the curve (AUC) value of 0.95, a sensitivity of 88.9%, and a specificity of 100%. In contrast, Dong et al. (10) included 36 ovarian cancer patients and 25 healthy controls in their study, utilizing PCR technology to detect the methylation status of the *SLIT2* promoter in ctDNA from peripheral blood in both groups. The results indicated that the incidence of *SLIT2* promoter methylation was 75% in ovarian cancer patients, while no *SLIT2* promoter methylation was detected in the control group, highlighting challenges in ​​analytical consistency​​.

Additionally, Li et al. (11) screened 493 CpG sites from over 3.3 million cfDNA methylation markers based on methylation differences and P-values, ultimately constructing the MethylBERT-EOC diagnostic model. This model demonstrated significant diagnostic efficacy for early-stage (I-II) epithelial ovarian cancer, achieving an AUC of 0.95, a sensitivity of 79.45%, and a specificity of 94.34%. Notably, when combined with CA125, the model’s diagnostic sensitivity for early-stage epithelial ovarian cancer increased from 82.51% to 89.62%, while maintaining a specificity of 93.55%. This represents a significant advancement in computational complexity and potential accuracy. However, the high development costs and the need for sophisticated bioinformatics infrastructure, in comparison to simpler PCR-based methylation assays, may restrict its immediate widespread application in clinical settings.

Cohen et al. (12) conducted a study involving 32 patients with high-grade serous ovarian cancer and 32 healthy controls for ctDNA sequencing. The findings revealed that genomic copy number variations were detected in 40.6% of ovarian cancer patients, with 38% of these being early-stage patients; conversely, only 6.25% of the healthy control group exhibited detectable plasma ctDNA copy number variations. The overall sensitivity of this detection method was approximately 41%, with a specificity of around 94%. Further analysis indicated a sensitivity of 33% for stage I/II patients and 44% for stage III/IV patients. Moreover, recent studies have shown that combining ctDNA concentration with integrity metrics significantly enhances the early diagnostic efficacy of ovarian cancer (13). This method is technically mature and cost-effective, making it suitable for targeted therapy selection. However, its utility in population screening is limited due to suboptimal sensitivity.

In addition, the multi-component integration strategy also shows great promise. The CancerSEEK method (14) facilitates early screening for eight common tumor types, including ovarian cancer, by detecting mutations in 16 genes within ctDNA and integrating this with an assessment of eight cancer-related proteins present in the patient’s blood. This method exhibits the highest sensitivity for ovarian cancer detection, reaching 98%, with a specificity exceeding 99%, thus significantly mitigating the risk of false-positive results. However, further research is necessary to evaluate the clinical applicability of this method and similar approaches. However, the translation of these findings into clinically available testing methods still requires large-scale prospective validation in both high-risk and general populations. Furthermore, ctDNA, as a novel biomarker, demonstrates commendable sensitivity and specificity in the diagnosis of ovarian cancer. When utilized alongside traditional biomarkers, it not only enhances diagnostic sensitivity but also preserves specificity, thereby facilitating the early detection of tumors. Existing evidence indicates that methylation-based detection achieves the best balance between sensitivity, specificity, and technological maturity, making it the most promising direction for early diagnosis at present. Future challenges lie in the large-scale prospective validation to transition it from a research tool to a standardized clinically available test, and to demonstrate its cost-effectiveness for screening in both high-risk and general populations.

### 4.2 Efficacy monitoring

The assessment of treatment response in ovarian cancer has traditionally relied on imaging and CA125 levels; however, these methods often exhibit delays and limitations.In ovarian cancer, changes in ctDNA concentration can dynamically reflect disease progression and have been shown to outperform traditional CA125 measurements (35). Furthermore, research indicates that ctDNA dynamics are associated with ovarian cancer patients’ responses to adjuvant chemotherapy and may predict treatment responses or disease progression (121).Research has shown that the dynamic changes in ctDNA levels are sensitive indicators for assessing treatment response. Over 96% of patients with high-grade serous ovarian cancer (HGSOC) exhibit alterations in the *TP53* gene, which is recognized as one of the most prevalent mutations in cancer (17, 18). Neoadjuvant chemotherapy (NACT), a treatment modality for cancer, impacts genes involved in the *TP53* signaling pathway (19). These mutations can serve as customizable markers for monitoring tumor growth and early changes, as well as for predicting response time and time to progression (TTP) (20). Analysis of ctDNA indicates that *TP53* mutations are valuable biomarkers for predicting responses to first-line therapy and for dynamically monitoring treatment responses in patients with HGSOC (21). Kim et al. (22) monitored *TP53* gene mutations in ctDNA before and after surgery in 28 patients with high-grade serous ovarian cancer. The results demonstrated that in patients who achieved satisfactory tumor debulking during surgery, *TP53* allele mutations decreased by 93%, whereas in patients with residual disease post-surgery, the decrease was only 74%. In patients with HGSOC, there is a baseline correlation between ctDNA levels and disease burden. However, a shorter TTP is associated with a decrease in *TP53* mutation allele frequency (MAF) of ≤60% following a single treatment cycle (23). This indicates that patients who may respond poorly to treatment can be identified very early in the therapeutic process.

High-sensitivity detection technology is the cornerstone for achieving precise monitoring. dPCR has become the ideal choice for longitudinal monitoring of known biomarkers due to its high specificity and sensitivity for specific mutations, such as TP53. On the other hand, NGS allows for a broader genomic analysis. For instance, the study conducted by Oikkonen et al. not only monitored the reduction in mutation frequency but also identified an increase in the frequency of TP53 mutations in the ctDNA of relapsed patients, effectively capturing the emergence of resistant clones (24).

These results highlight the significant role of plasma ctDNA testing in evaluating the efficacy of treatment for ovarian cancer. For patients with ovarian cancer, timely assessments of treatment efficacy are crucial for selecting appropriate subsequent treatment regimens. Despite its immense potential, the core obstacle to integrating ctDNA monitoring into routine clinical practice lies in the lack of standardized molecular response thresholds.

### 4.3 Treatment guidelines

*BRCA1/2* germline mutations are the most significant genetic risk factors for epithelial ovarian cancer, occurring in 6–15% of women diagnosed with this disease. The treatment of ovarian cancer has entered the era of precision medicine, particularly with the application of poly (ADP-ribose) polymerase (PARP) inhibitors, which has profoundly changed the treatment landscape for patients with BRCA mutations. In clinical practice, PARP inhibitors are employed as maintenance therapy for ovarian cancer patients harboring *BRCA* gene mutations (122). However, the occurrence of reverse mutations in *BRCA* genes may diminish the efficacy of PARP inhibitors and potentially lead to resistance against these drugs (123, 124). Previous studies have predominantly concentrated on reverse mutations in circulating ctDNA from ovarian cancer patients, aiming to enhance the guidance for related drug therapies in clinical settings.

In HGSOC at stages III and IV, dynamic analysis of *BRCA1/2* ctDNA can detect reversal mutations or mutations in *BRCA1* and *TP53*, thereby predicting patients’ responses to PARP inhibitor therapy (26). Additionally, somatic reversal mutations or intragenic deletions that restore *BRCA1* or *BRCA2* function can confer resistance to PARP inhibitors or platinum-based chemotherapy in patients harboring germline *BRCA1* or *BRCA2* mutations (27). ctDNA sequencing analysis can identify potential *BRCA1/2* reversal mutations in ovarian cancer patients (28), facilitating the screening of patients who are suitable for PARP inhibitor therapy (29, 30). Christie et al. (31) identified five cases of BRCA gene reversal mutations in tumor DNA from 30 patients with high-grade serous ovarian cancer through NGS analysis of *TP53*, *BRCA1*, and *BRCA2*. Among these, three patients exhibited the same reversal mutation in their ctDNA, with a detection sensitivity of 60%. These three patients developed resistance to platinum-based drugs or PARP inhibitors but demonstrated favorable responses to gemcitabine and bevacizumab therapy. Lin et al. (32) conducted ctDNA *BRCA1* and *BRCA2* reversal mutation testing in 78 patients with epithelial ovarian cancer prior to PARP inhibitor therapy, identifying eight patients with reversal mutations, whose progression-free survival (PFS) was significantly lower than that of patients without such mutations. In another study, ctDNA *BRCA1* and *BRCA2* reversal mutations were detected in 112 ovarian cancer patients treated with PARP inhibitors, with eight patients acquiring reversal mutations. Among these, four patients experienced disease progression after a median follow-up of 3.5 months, while the remaining four had reversal mutations detected at the time of disease progression. An additional analysis of 54 ovarian cancer patients identified three cases of *BRCA* reversal and demonstrated that mutation heterogeneity improved progression after PARP inhibitors, suggesting non-exclusive acquired resistance mechanisms such as upregulation of survival pathways (41%), replication fork stability (34%), homologous recombination repair restoration (28%), loss of targets (10%), and drug efflux (3%) (33). Additionally, a study collected 69 plasma samples from 135 ovarian cancer patients before and after systemic therapy. The results revealed that 60% of patients exhibited high methylation of the *BRCA1* promoter during treatment, while 24% reverted from this high methylation state. This finding suggests that ctDNA testing plays a significant role in identifying treatment-resistant clones (34).

These results indicate that the analysis of ctDNA highlights various acquired resistance mechanisms, which will assist in treatment selection and resistance monitoring. The current challenge lies in how to translate the detection results into specific clinical actions, particularly in determining the optimal treatment plan after identifying resistant mutations. Furthermore, the sensitivity of ctDNA testing is not 100%, and negative results do not completely rule out resistance. Future prospective trials are needed to clarify how to best integrate ctDNA into the clinical decision-making pathways for resistant ovarian cancer.

### 4.4 Prognosis assessment

Early detection of recurrence is crucial for guiding treatment decisions and reducing patient turnaround time, ultimately improving clinical outcomes. The detection of MRD after surgery or radical treatment is one of the most commonly used methods for prognostic assessment, in which ctDNA demonstrates superior performance compared to traditional methods.

Research indicates that ctDNA analysis exhibits higher sensitivity and specificity than CA-125 in detecting recurrence, typically identifying recurrences an average of 10 months earlier than imaging studies (35). Additionally, recent findings suggest that elevated levels of somatic mutations in ctDNA may correlate with decreased clinical treatment benefits and poorer PFS or overall survival (OS) (36, 37). Variations in ctDNA methylation levels during or after treatment generally serve as predictive indicators of patient prognosis. Advanced-stage patients are more likely to exhibit detectable ctDNA mutations prior to surgery and adjuvant chemotherapy, with these mutations being associated with poorer OS post-surgery, thereby establishing ctDNA as an independent predictor of PFS and OS (38).

ctDNA can achieve quantitative risk stratification. The study further confirms that ovarian cancer patients who underwent neoadjuvant surgery and exhibited higher ctDNA mutation copy numbers in plasma (>10 mutation copies/mL) had significantly lower OS compared to those with lower copy numbers (<10 mutation copies/mL) (125).

In a prospective study, researchers conducted serial ctDNA testing on 23 ovarian cancer patients to evaluate its potential in predicting disease recurrence after primary tumor resection. The results indicated that ctDNA achieved a sensitivity of 91% in predicting tumor recurrence (126). Additionally, studies have shown that detectable levels of ctDNA in plasma can predict disease recurrence an average of 7 months before CT scans (70).

Vitale et al. analyzed serum samples from 20 patients with high-grade serous ovarian cancer following their initial debulking surgery to investigate *TP53*-mutated ctDNA sequences. They found that patients with suboptimal debulking (67%) exhibited a higher proportion of *TP53*-mutated ctDNA compared to those with optimal debulking (45%) (127). Research presented at the 2024 ASCO Annual Meeting confirms that ctDNA is a powerful prognostic tool for guiding MRD monitoring in advanced ovarian cancer (128). Its sensitivity for detecting residual lesions (21.2%) is significantly higher than that of CA125 (1.3%). Patients with negative ctDNA results have an extremely low risk of recurrence (negative predictive value of 98.3%), while those with positive ctDNA results have a recurrence risk 57.5 times greater than that of negative patients, which is markedly higher than the 6.3-fold increase in risk associated with positive CA125 results. Consequently, monitoring postoperative MRD status and continuous ctDNA assessment during maintenance therapy are optimal precision management tools for predicting recurrence risk and guiding clinical treatment decisions, providing critical evidence for the precision treatment of ovarian cancer. The core challenge lies in determining the ctDNA threshold that triggers clinical intervention.

In summary, dynamic monitoring of ctDNA at the molecular level holds significant promise in assessing the prognosis of ovarian cancer patients. Future prospective studies are warranted to further validate whether ctDNA can serve as a novel biomarker for the diagnosis and prognosis of ovarian cancer.

## 5 Application of ctDNA in cervical cancer

### 5.1 Early diagnosis and screening

Cervical cancer is primarily caused by persistent infection with high-risk human papillomavirus (HPV), with subtypes 16 and 18 accounting for approximately 70% of cases (129). Currently, the most commonly used methods for cervical cancer screening and diagnosis include liquid-based cytology and HPV-DNA testing. ctDNA analysis provides a new dimension for screening, particularly based on HPV ctDNA analysis. Studies have shown that in cervical cancer, the HPV viral genome can integrate into the tumor cell genome or free DNA (130), and can be detected in the plasma of HPV-associated cervical cancer patients using different PCR techniques (131).

HPV ctDNA has a natural high specificity due to its viral specificity. Gu et al. (39) established that HPV ctDNA serves as a specific diagnostic marker for cervical cancer, exhibiting moderate sensitivity (0.27, 95% CI: 0.24–0.30) and high specificity (0.94, 95% CI: 0.92–0.96), reflecting its high diagnostic accuracy. Multiple studies (40, 41) have identified that HPV ctDNA can be detected in blood samples from HPV-positive cervical cancer patients prior to treatment, while it is rarely found in HPV-positive cervical precancerous lesions or in patients without cervical lesions. Mittelstadt et al. (42) investigated HPV ctDNA in the plasma of cervical cancer patients and found a significant correlation between tumor burden and HPV ctDNA levels. Additionally, a quantitative study utilizing ddPCR technology revealed that HPV ctDNA levels in cervical cancer patients are associated with clinical stage and tumor size. Even in the subclinical stage, patients with HPV-related invasive cancer can have detectable HPV ctDNA in their plasma, with levels closely correlated to tumor dynamics (43).

Currently, ctDNA testing has been incorporated into the 2022 NCCN guidelines for cervical cancer, indicating that ctDNA can be considered for whole-genome analysis when tumor tissue samples are unavailable. Its future role may involve triaging women who test positive in primary HPV screening, identifying high-risk populations with integrated and transformative infections, thereby optimizing the referral strategy for colposcopy and improving screening efficiency.

### 5.2 Efficacy monitoring

Despite significant differences in ctDNA among different patients, the dynamic changes of ctDNA over time in individual patients are closely related to treatment response and disease burden. Therefore, ctDNA can serve as a specific biomarker for assessing treatment efficacy. The monitoring of treatment effects in cervical cancer can also benefit from the dynamic analysis of ctDNA, as its changes can reflect the tumor’s response to radiotherapy and chemotherapy in real-time.

HPV ctDNA is an ideal biomarker for monitoring the tumor burden associated with viral infections. Kim et al. used NGS to monitor the treatment response of 25 cervical cancer patients following radical radiotherapy, employing HPV16/18 and 67 cancer gene target capture libraries. They assessed the efficacy of HPV ctDNA in comparison to traditional treatment monitoring methods, such as tumor markers and magnetic resonance imaging (MRI). The tumor markers evaluated included squamous cell carcinoma antigen (SCC) and carcinoembryonic antigen (CEA). HPV ctDNA was quantified by calculating the ratio of HPV read counts to total read counts (human reads + HPV reads). The results indicated that, in most cases, both tumor marker levels and HPV ratios decreased as tumor volume diminished. Notably, the HPV ratio exhibited greater sensitivity in detecting temporal changes between the second and third patient visits. In two patients with distant metastasis, the HPV ratio was significantly elevated compared to tumor markers. Conversely, in patients without evidence of disease, the HPV ratio decreased while tumor marker levels increased. These findings suggest that the HPV ctDNA ratio, based on targeted NGS, may serve as an effective tool for treatment monitoring and prognostic assessment (44).

Conversely, Li et al. (45) prospectively recruited 16 patients with locally advanced cervical cancer who required neoadjuvant chemotherapy. Through next-generation sequencing (NGS) analysis of circulating tumor DNA (ctDNA) and tumor tissue DNA (ttDNA) from these patients, mutations in *PBRM1, SETD2*, and *ROS1* were frequently detected in both ctDNA and ttDNA of non-responders. Among these mutations, *PBRM1* mutations were linked to poorer survival outcomes in patients. *In vitro* experiments demonstrated that knocking down *PBRM1* enhanced *STAT3* signaling in cervical cancer cells, thereby promoting resistance to cisplatin. These findings suggest that *PBRM1* mutations may serve as potential ctDNA biomarkers for drug resistance in patients undergoing neoadjuvant chemotherapy. This indicates that ctDNA analysis can not only monitor tumor burden but also reveal mechanisms of resistance, providing molecular evidence for timely adjustments to treatment regimens.

Therefore, in contrast to traditional imaging examinations and pathological biopsies, ctDNA can more effectively monitor treatment responses and evaluate treatment efficacy during cervical cancer therapy. Additionally, it allows for dynamic adjustments to treatment plans based on fluctuations in its concentration levels, thereby facilitating individualized management and precision treatment for patients.

### 5.3 Treatment guidelines

Traditional treatment for cervical cancer primarily relies on surgery combined with radiotherapy and systemic chemotherapy (132). ctDNA offers the potential for precision therapy by identifying actionable genetic mutations. Numerous studies have demonstrated that monitoring treatment via ctDNA is a sensitive method compared to traditional tissue biopsy or imaging techniques (133).

Charo et al. (47) analyzed ctDNA sequencing results from 13 cervical cancer patients alongside other gynecological tumor patients. In cervical cancer patients, *PIK3CA* emerged as the most frequently mutated gene. The study results indicated that in multivariate analysis, a higher maximum mutated allele frequency of ctDNA was associated with poorer OS (HR = 1.91, P = 0.03). In contrast, patients who received treatment aligned with ctDNA alterations exhibited significantly improved OS (HR = 0.34, P = 0.007), while those who did not receive matched treatment did not show such an association. This study suggests that the detection of ctDNA levels can identify gene mutations with potential value for diagnosis, prognosis, and tumor monitoring, thereby guiding treatment decisions. Additionally, Zhang et al. (48) conducted NGS testing covering 1,020 genes on plasma samples from 126 cervical cancer patients in China. The results indicated that *PIK3CA* was the most commonly mutated gene in cervical cancer (approximately 30%), consistent with the detection results of *PIK3CA* in cervical cancer tissue. This finding further confirms the correlation between plasma ctDNA and mutated genes in tumor tissue, providing a basis for selecting targeted therapies based on ctDNA mutations.

Lalondrelle et al. (49) recently conducted a study using an NGS technique called “panHPV detection” to detect HPV ctDNA and evaluate treatment responses in patients with locally advanced cervical cancer. The test successfully identified recurrence in three patients, despite imaging studies indicating complete clearance, with HPV ctDNA being detected three months post-treatment. Furthermore, four patients exhibiting partial clearance on imaging, who did not show HPV ctDNA at the three-month follow-up, displayed no signs of recurrence. These findings suggest that panHPV ctDNA detection holds significant potential for assessing treatment response and predicting recurrence in cervical cancer patients following chemoradiotherapy.

Currently, targeted treatment options for cervical cancer remain relatively limited. The guiding role of ctDNA is more evident in identifying patients who could benefit from specific targeted clinical trials. In the future, as more targeted drugs are developed, the guiding value of ctDNA will further increase. However, further prospective studies are needed to validate the guidance on the timing and selection of treatment regimens.

### 5.4 Prognosis assessment

Most cervical cancer patients require regular follow-up examinations within five years after completing treatment. These follow-up procedures primarily involve monitoring for recurrence and metastasis of cervical cancer, evaluating adverse reactions that may have occurred during treatment, and conducting comprehensive systemic examinations. Circulating tumor DNA testing has the advantage of being repeatable in terms of sample collection and processing, enabling dynamic monitoring of tumor progression in patients. Multiple studies have shown that ctDNA can be used to assess the efficacy of radiotherapy, chemotherapy, and other treatment regimens. Notably, the detection levels of HPV ctDNA not only serve to evaluate treatment outcomes but are also closely associated with cervical cancer prognosis; however, the results of these tests are influenced by various factors (134).

Multiple studies have confirmed the prognostic value of ctDNA. A study conducted by Jeannot et al. revealed that HPV ctDNA was detected in 63% of patients prior to treatment, with ctDNA levels positively correlated to higher FIGO stages and para-aortic lymph node involvement. Furthermore, complete clearance of HPV ctDNA at the conclusion of treatment was significantly associated with extended progression-free survival, suggesting that HPV ctDNA detection can serve as an effective biomarker for predicting cervical cancer recurrence (50). Cabel et al. (51) analyzed blood and tumor samples from 55 HPV-positive patients with locally advanced cervical cancer who underwent chemoradiotherapy, finding that positive HPV ctDNA detection was linked to lower DFS (P = 0.048) and OS (P = 0.0013). This indicates that residual HPV ctDNA at the end of chemoradiotherapy may assist in identifying patients at risk of subsequent recurrence. In a recent study, Han et al. (52) compared the accuracy of FDG-PET and ddPCR in predicting plasma HPV ctDNA levels in patients with locally advanced cervical cancer. Through serial sampling, post-treatment plasma HPV ctDNA levels were shown to predict metastasis. The study reported that patients with undetectable HPV ctDNA levels in plasma after treatment were associated with higher PFS compared to those with detectable HPV DNA levels. Collectively, these studies indicate that ctDNA possesses significant prognostic predictive value for cervical cancer.

Recently, Chung et al. (46) used ddPCR to assess the efficacy of *PIK3CA* mutation detection in cervical cancer patients. In plasma ctDNA samples collected prior to treatment from 177 patients with invasive cervical cancer, two *PIK3CA* mutations (p.E545K and p.E542K) were identified. The results indicated that 22.2% of patients had at least one *PIK3CA* mutation in their plasma ctDNA. Notably, the study found that the *PIK3CA* mutation status in plasma ctDNA was significantly associated with reduced median tumor size, disease-free survival, and overall survival. A retrospective clinical study by Tian et al. (53) found that ctDNA could detect recurrent metastatic cervical cancer patients with any mutations in *PIK3CA, BRAF, GNA11, FBXW7*, and *CDH1*, whose PFS and OS were significantly shorter than those without detectable mutations.

The recent CALLA study reveals that in patients with locally advanced cervical cancer (LACC), the application of ultra-high-sensitivity personalized ctDNA detection based on whole-genome sequencing (NeXT Personal) demonstrates exceptional sensitivity, evidenced by a baseline detection rate of 99%. The findings indicate that elevated baseline ctDNA levels correlate with an increased risk of disease progression. Notably, the detection of ctDNA during treatment (cycle 3) and post-treatment (6 months) serves as a robust predictor of significantly heightened recurrence risk, with hazard ratios (HR) as low as 0.04–0.05. Furthermore, ctDNA clearance is closely linked to improved PFS, with the median PFS not yet reached. Although the combination therapy with durvalumab (in contrast to radiotherapy and chemotherapy alone) enhances ctDNA clearance, particularly in the PD-L1-high expression subgroup, no significant difference in disease progression risk was observed between the two groups when evaluated based on ctDNA status. Consequently, this study primarily establishes the significance of ctDNA as an independent prognostic biomarker, thereby supporting its future application in guiding risk stratification and treatment decisions for LACC patients (54).

In conclusion, ctDNA monitoring is pivotal in prognostic assessment and recurrence surveillance during the treatment of cervical cancer patients. It functions not only as a residual tumor marker at the conclusion of treatment but also as a sensitive biological indicator for monitoring recurrence during follow-up.

## 6 Application of ctDNA in endometrial cancer

### 6.1 Early diagnosis

Although the overall prognosis for endometrial cancer is generally favorable, approximately 30% of cases are diagnosed at an advanced stage with invasive metastasis, leading to a poorer prognosis. Early diagnosis can significantly improve patient outcomes (135). However, there is currently no established screening protocol for endometrial cancer, either in the general population or among specific high-risk groups (136). Therefore, identifying more sensitive and specific screening markers is crucial, and ctDNA has emerged as a promising biomarker for endometrial cancer.

Current studies have validated the feasibility of screening early-stage patients by detecting somatic mutations associated with endometrial cancer in ctDNA. Feng et al. (55) found that for high-risk endometrial cancer, the mutated genes detected in ctDNA were highly consistent with those in baseline tumor tissue. Bolivar et al. (56) identified four common genes associated with endometrial cancer (*CTNNB1, KRAS, PTEN, PIK3CA*) and detected at least one mutation in 94% of patients, with mutations found in the plasma of 18% of early-stage patients. Additionally, enhancing the efficacy of early screening can be achieved by detecting ctDNA fragment length, copy number, or by combining ctDNA detection with other protein biomarkers. A study reported by ASCO in 2018 also indicated that the sensitivity of ctDNA detection for patients with endometrial cancer is less than 10%, while its specificity reaches as high as 95% (57).

Furthermore, a study combined ctDNA profiling with tumor-induced platelets (TEPs) to test 71 genes (58). This method utilizes TEPs for preliminary diagnosis to distinguish between healthy women and endometrial cancer patients, followed by ctDNA analysis for histological diagnosis. This approach provides valuable support for endometrial cancer diagnosis, achieving a sensitivity of 77.8% and an accuracy rate of 68.7%. Recent research has further indicated that the detection of microsatellite instability (MSI) and ctDNA in endometrial aspirates can function as a diagnostic tool for minimally invasive subtypes of endometrial cancer and can be utilized to monitor patients’ responses to immune checkpoint inhibitors (59, 60).

In summary, ctDNA not only serves as a diagnostic biomarker for endometrial cancer, but research on molecular subtyping guided by it is also likely to become an important future research direction, laying the foundation for the precise management of endometrial cancer.

### 6.2 Efficacy monitoring

The heterogeneity of endometrial cancer can lead to both congenital and acquired drug resistance, resulting in poor responses to conventional chemotherapy and targeted therapies. In such cases, characterizing tumor heterogeneity and monitoring tumor response to drugs in real-time can facilitate more accurate predictions of specific clinical outcomes at diagnosis, thereby enabling the development of the most appropriate treatment plan.

The levels of ctDNA are correlated with tumor burden and treatment response. Studies have shown that ctDNA testing is primarily conducted in high-risk endometrial cancer patients (55, 137). Ashley et al. (61) found that ctDNA levels are associated with endometrial cancer staging, and continuous measurement can reflect treatment response, disease progression, and recurrence. In over 90% of cases, tumor somatic mutations were accurately identified in ctDNA, and the presence of baseline or postoperative ctDNA was significantly associated with reduced PFS. Carlos et al. (62) used ddPCR technology to measure ctDNA levels in 60 endometrial cancer patients. The results indicated that 56.3% of high-risk tumor patients were ctDNA-positive at the time of surgery, whereas only 15.8% of low-risk tumor patients were ctDNA-positive. Furthermore, endometrial cancer patients with higher ctDNA levels and deeper myometrial invasion exhibited a higher tumor burden, suggesting that ctDNA can be employed to monitor tumor burden.

Silveira et al. (63) reported that ctDNA detection results for endometrial cancer exhibited high consistency (93%) with the clinically established reference pentablot method, and its dynamic changes during treatment were closely related to clinical response. Some researchers have utilized ddPCR to detect *PIK3CA* or *KRAS* mutations in ctDNA to explore the association between ctDNA and endometrial cancer (64). The results demonstrated that ctDNA was significantly associated with the histopathological type, stage, and grade of endometrial cancer and served as an independent risk factor for lymph node metastasis. Collectively, these studies indicate that ctDNA is a promising dynamic biomarker for assessing tumor burden and evaluating treatment efficacy.

In summary, the absence of specific biomarkers for monitoring disease activity in endometrial cancer underscores the significant clinical value of endometrial cancer-specific ctDNA testing. This approach is particularly beneficial for monitoring recurrence and guiding future treatment decisions.

### 6.3 Treatment guidelines

The treatment decisions for endometrial cancer, particularly regarding the use of immunotherapy, are highly dependent on molecular subtyping.

ctDNA plays a crucial role in the treatment of endometrial cancer. A large-scale Phase II KEYNOTE-158 study (65) demonstrated that PD-1 inhibitors exhibit superior efficacy in patients with microsatellite instability-high (MSI-H) or mismatch repair-deficient (dMMR) tumors. Additionally, ctDNA can effectively identify MSI-H status in recurrent endometrial carcinoma (66). Clinical guidelines recommend determining the MSI status of endometrial cancer patients to guide the use of PD-1 inhibitors based on ctDNA (67). In the DUO-E study of advanced or recurrent endometrial cancer, dynamic ctDNA monitoring revealed that 80% of patients had detectable ctDNA at baseline, and its presence was associated with shorter PFS and was a poor prognostic factor; a decrease in ctDNA levels during treatment was strongly associated with significantly improved prognosis. The study specifically demonstrated that in patients with proficient mismatch repair (pMMR), the combination of olaparib and durvalumab maintenance therapy significantly enhanced ctDNA clearance rates during the maintenance phase (48% vs. 17% with single-agent maintenance), elucidating the potential mechanism behind the improved efficacy of this regimen in pMMR patients (68, 69).

In summary, ctDNA, as a real-time and sensitive biomarker, shows considerable promise in evaluating treatment response, predicting recurrence risk, and guiding personalized treatment decisions, thereby advancing precision therapy for endometrial cancer.

### 6.4 Prognosis assessment

High-risk endometrial cancer is characterized by elevated rates of metastasis and recurrence. Patients who experience recurrence tend to have poorer prognoses. Consequently, monitoring for recurrence and assessing prognosis are critical for developing appropriate individualized treatment plans. This approach ensures that patients at high risk of recurrence receive timely adjuvant therapy while avoiding unnecessary treatments for low-risk patients.

Pereira et al. (70) hypothesized that ctDNA serves as an independent predictor of survival in patients with endometrial cancer. Their research demonstrated that regular monitoring of ctDNA levels allows for earlier diagnosis of disease recurrence compared to traditional CT scans. Furthermore, post-treatment monitoring of ctDNA levels provides novel indicators for survival prediction. In a study involving nine high-risk endometrial cancer patients (55), ctDNA was detectable in 67% of baseline plasma samples, with significantly elevated levels of both cfDNA and ctDNA observed in recurrent patients compared to non-recurrent patients. The study also evaluated the correlation between ctDNA and tumor recurrence, revealing that postoperative ctDNA levels exhibited a sensitivity of 100% and a specificity of 83.3% for predicting tumor recurrence, with a Kappa index of 0.769. These findings indicate a substantial consistency between ctDNA detection and postoperative tumor recurrence, suggesting that ctDNA is superior to CA125 or HE4 in detecting tumor recurrence (71).

ctDNA can provide a significant lead time. Additionally, a study conducted in China involving 46 patients with endometrial cancer found that recurrent patients exhibited negative ctDNA levels preoperatively, which turned positive during postoperative follow-up, with clinical recurrence confirmed one month later (3). A recent small-sample study (66) also indicated that ctDNA can detect recurrence or progression of endometrial cancer approximately 2.5 months (ranging from 1 to 8 months) earlier than traditional imaging techniques.

In patients diagnosed with stage I endometrial cancer who subsequently relapsed, elevated ctDNA levels signaled disease progression, which was confirmed by imaging techniques. Additionally, ctDNA can accurately reflect the response to radiotherapy in patients with recurrent endometrial cancer undergoing chemotherapy. A recent real-world study demonstrated that ctDNA testing holds significant prognostic value in patients with advanced endometrial cancer. Patients with ctDNA positivity, particularly those carrying *TP53* mutations, exhibited significantly poorer survival outcomes (median real-world overall survival [rwOS]: 71.2 months vs. 108 months; hazard ratio [HR]: 0.75; 95% confidence interval [CI]: 0.59–0.96; P = 0.02). When *TP53* co-mutates or co-amplifies with other genes, it further exacerbates survival risk (rwOS: 66.4 months vs. 78.1 months; HR: 0.66; 95% CI: 0.47–0.92; P = 0.016). Genomic profiling analysis based on ctDNA revealed key driver mutations, and longitudinal monitoring can dynamically reflect tumor evolution and early progression. These findings support the integration of ctDNA, particularly *TP53* status, into the management of endometrial cancer for risk stratification, prognostic assessment, and treatment monitoring (72). Notably, *TP53* mutations also possess therapeutic predictive value; subsequent analysis of the GOG-86P trial showed that in advanced or recurrent patients, the *TP53* mutation subgroup (44%) receiving bevacizumab plus chemotherapy (paclitaxel/carboplatin) had significantly longer survival compared to the temsirolimus regimen (median PFS: 12.5 months vs. 8.2 months; median OS: 29.1 months vs. 22.0 months), while wild-type patients did not benefit from this combination (73).

In summary, ctDNA is closely related to the prognosis of endometrial cancer and can serve as a real-time monitoring biomarker for assessing prognosis and detecting recurrence. Integrating ctDNA, particularly the *TP53* status, for risk stratification, prognostic evaluation, and treatment monitoring is an important direction for the future.

## 7 Discussion

As research into ctDNA in gynecological tumors continues to deepen, ctDNA is emerging as a non-invasive and dynamic biomarker with broad application prospects in early diagnosis, treatment monitoring, detection of minimal residual disease, and prognosis assessment. An analysis of existing studies reveals that the potential of ctDNA in the field of gynecological tumors has garnered widespread attention and has, to some extent, driven changes in clinical practice. Although the focus of research varies across different tumor types, all studies provide a robust foundation for the application of ctDNA. The core commonality lies in its ability to provide an earlier and more sensitive dynamic reflection of tumor burden compared to traditional imaging and serum protein biomarkers. This provides highly consistent clinical information for monitoring the efficacy of gynecological tumors, detecting MRD, and predicting recurrence. Postoperative or post-treatment ctDNA positivity serves as a powerful, common predictor of poor prognosis, and its detection provides a critical window for intervention, holding immense promise as a universal biomarker for guiding adjuvant therapy decisions. However, the focus and maturity of its application vary significantly depending on the biological characteristics of each cancer, revealing distinct specificities. In ovarian cancer, research concentrates on leveraging *TP53* mutations for treatment response assessment and identifying *BRCA1/2* reversion mutations to guide PARP inhibitor therapy and unravel resistance mechanisms. In cervical cancer, its viral etiology makes the quantitative monitoring of HPV ctDNA a unique and highly specific approach for assessing treatment response and viral-associated tumor burden. For endometrial cancer, the application specificity focuses on detecting MSI status to identify populations most likely to benefit from immunotherapy.

As a potential screening tool for early cancer detection, ctDNA holds significant promise but faces numerous challenges. Currently, the field of ctDNA analysis lacks standardized procedures for collection, extraction, and amplification, which complicates the effective translation of detection results across different clinical settings. It is well known that gene methylation and somatic mutations accumulate in plasma with age. We need to distinguish whether the molecular levels detected in plasma originate from ctDNA or from normal aging and degenerative tissue cells. Furthermore, clonal hematopoiesis poses a potential barrier, as early malignant tumors may release only a small amount of ctDNA, limiting sensitivity and increasing the risk of false negatives, while clonal hematopoiesis may obscure ctDNA detection in elderly patients (138). Additionally, changes in molecular levels caused by factors such as inflammation must also be considered (2). Studies have shown that the amount of ctDNA released by primary tumors and metastatic sites varies significantly (139), a phenomenon that may limit the clinical applicability of ctDNA in metastatic diseases. Multiple studies have confirmed that the detectable levels of ctDNA in plasma are not only related to tumor type but are also influenced by factors such as tumor stage, disease burden, and the presence of metastasis. A study on ctDNA detection in advanced cancer found that ctDNA was undetectable in up to 15% of advanced cancer cases. The type and stage of cancer also influence detection rates; over 75% of patients with advanced pancreatic cancer, ovarian cancer, colorectal cancer, bladder cancer, gastroesophageal cancer, breast cancer, melanoma, hepatocellular carcinoma, and head and neck cancer had detectable ctDNA. However, the proportion of ctDNA detected in primary brain cancer, kidney cancer, prostate cancer, or thyroid cancer was less than 50% (140). Therefore, future research should prioritize the evaluation of the technical stability and reproducibility of ctDNA as a biomarker within and across laboratories by implementing standardized procedures.

On the other hand, ctDNA is primarily suitable for molecular analysis, DNA sequencing, and the identification of mutations and variations. In contrast, tissue biopsy not only permits histological interpretation of tumors but also identifies significant non-DNA alterations, such as proteins and hormone receptors. These non-DNA biomarkers, in conjunction with molecular sequencing results, play a crucial role in formulating treatment strategies. Thus, tissue biopsy remains the gold standard for molecular testing and diagnosis. In the context of early cancer detection, the amount of ctDNA in patients’ blood can be exceedingly low, making it challenging to measure and detect such small quantities, which may also lead to false negatives. ctDNA testing technology must demonstrate extremely high sensitivity prior to the manifestation of clinical symptoms to detect trace amounts of ctDNA in a patient’s serum. Simultaneously, if ctDNA testing is to be utilized as a screening tool, its design must ensure sufficient specificity, maintaining a low false-negative rate among a large asymptomatic population, thereby enhancing the feasibility of the test and its public acceptance. Most ctDNA biomarkers for gynecological tumors are often detected in other types of cancers, which may lead to confusion and interference in the diagnosis of gynecological tumors, especially regarding their accurate prediction. Therefore, more experiments are needed to identify specific ctDNA for gynecological tumors to improve the detection rate of early screening.

## 8 Conclusion

In summary, ctDNA, as an emerging molecular biomarker for tumors, shows significant potential for application in the early diagnosis and treatment of gynecological tumors. Despite facing numerous challenges, the continuous advancement of technology and the in-depth development of clinical research suggest that ctDNA is poised to become a crucial tool for the precise diagnosis and treatment of gynecological tumors, thereby providing substantial support for the realization of personalized medicine. Future research should prioritize multicenter, large-scale prospective clinical trials to accumulate more evidence-based medical data and further enhance the clinical application value of ctDNA.
